# Giant cell tumor of bone: A single center study of 115 cases

**DOI:** 10.1016/j.jbo.2022.100417

**Published:** 2022-02-17

**Authors:** Niklas Deventer, Tymoteusz Budny, Georg Gosheger, Anna Rachbauer, Jan Puetzler, Jan Christoph Theil, Dmytrii Kovtun, Marieke de Vaal, Nils Deventer

**Affiliations:** a Department of Orthopedics and Tumororthopedics, University Hospital Muenster, Albert-Schweitzer-Campus 1, 48149 Muenster, Germany; b Department of General Paediatrics, University Children's Hospital Muenster, Albert-Schweitzer-Campus 1, 48149 Muenster, Germany

**Keywords:** GCTB, Giant cell tumor of bone, Giant cell tumor, Giant cell tumor of bone, Intralesional curettage, Denosumab

## Abstract

**Background:**

Giant cell tumor of bone (GCTB) is a locally aggressive bone tumor that represents about 4–5% of all primary bone tumors. It is characterized by aggressive growth, possible recurrence after surgical treatment and, in rare cases, metastasis. Surgical management is the primary treatment and may include intralesional curettage with adjuvants or, in rare cases, wide resection. In recent years the monoclonal antibody denosumab has been introduced as a potential (neo-)adjuvant systemic treatment option for patients with borderline resectable or unresectable lesions. Currently several studies reported that the use of denosumab prior to curettage possibly increase the risk of local recurrence.

**Methods:**

In this retrospective study we reviewed 115 cases of GCT with a mean follow-up of 65.6 (24–404) months who underwent a surgical treatment with or without preoperative denosumab therapy in our institution. Potential risk factors for LR and complications were analyzed.

**Results:**

The study includes 47 male (40.9%) and 68 female (59.1%) patients with a mean age of 33.9 (10–77) years and a mean follow-up of 65.6 (24–404) months. Denosumab was used in 33 (28.7%) cases, in 14 cases (12.2%) in a neoadjuvant setting and in 17 cases preoperatively before re-curettage (14.8%) after LR. In 105 cases (91.3%) an intralesional curettage was performed. The overall LR rate was 47.8% (55 cases). Patients who underwent intralesional curettage and bone cement augmentation without neoadjuvant denosumab treatment had LR in 42.2% (38/90) of the cases. Patients who underwent neoadjuvant denosumab treatment prior to curettage had LR in 28.6% (4/14). Re-recurrence was frequent in patients with neoadjuvant denosumab treatment who had LR after initial curettage (50%, 8/16). After wide resection and endoprosthetic replacement one case (20%) of local recurrence was detectable (1/5 cases).

**Conclusions:**

GCTB recurs frequently after intralesional curettage and cement augmentation. While denosumab is a potential (neo-)adjuvant treatment option that might be used for lesions that are difficult to resect, surgeons should be aware that LR is still frequent.

## Highlights

• Giant cell tumor of bone (GCTB) is a rarely metastasizing, locally aggressive tumor.
• GCTB recurs frequently after intralesional curettage.
• Denosumab is a potential neoadjuvant treatment option for borderline resectable lesions.

## 1 Introduction

**Fig. 1:**
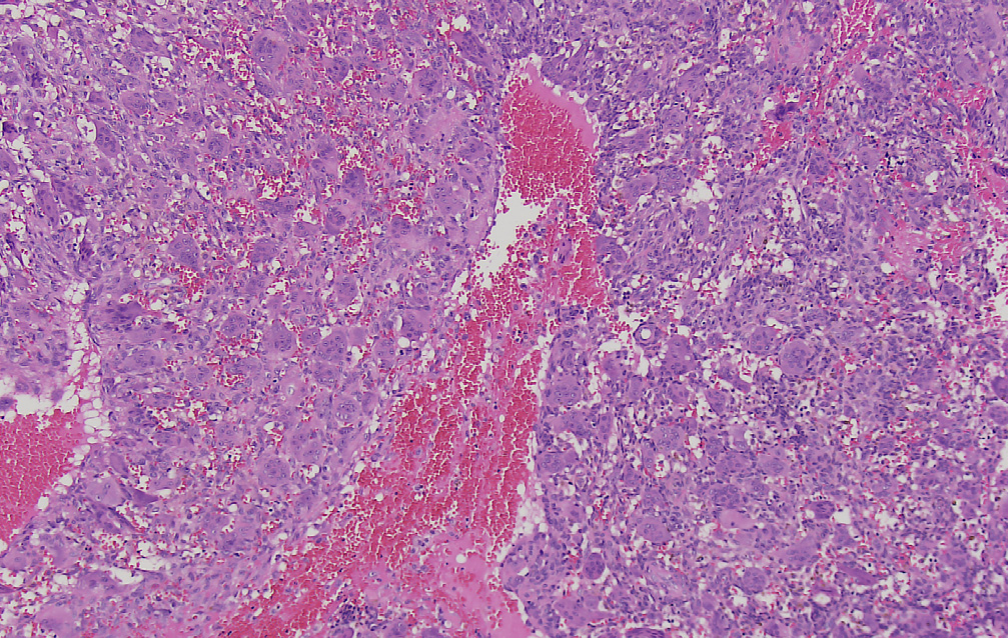
Microscopic image of a giant cell tumor of bone, H&E stain. (mononuclear) cells.

The giant cell tumor of bone (GCTB) is a rare, locally aggressive bone tumor that accounts for approximately 4–5 % of all primary bone tumors [1]. Females are affected more often than males [2]. It occurs primarily in patients between 30 and 40 years old [3]. GCTB may show pulmonary metastases; malignant transformation of the tumor is described in rare cases [4]. The most common sites of localization are the distal femur, the proximal tibia and the distal radius [5]. Clinical manifestations include pain, local swelling, effusion and limited motion of the affected joint. In radiographs GCTB occurs as osteolytic lesion in the epiphysis, often with joint affection. The diagnosis can be stated after open or needle biopsy of the lesion. Histologically the tumor contains diffusely spread multinucleated giant cells and neoplastic mononucleated fusiform-shaped stromal cells (Fig. 1) [6]. The multinucleated giant cells express receptor activator of nuclear factor kappa B (RANK) which leads to activation of osteoclasts and progressive osteolysis.

Surgical treatment is the standard treatment for GCTB [2]. Intralesional curettage with or without the use of adjuvants is the most common treatment [2], [7], [8]. Resulting bone defects can be filled with autologous bone, bone substitute or bone cement. Local adjuvants such as phenol or hydrogen peroxide can be used for irrigation of the tumor cavity after curettage [2], [7]. Nevertheless, a wide resection is an option for selected patients. There are also reports about the use of radiofrequency ablation or radiation with or without combined surgical treatment [9]. Reported local recurrence rates vary between less than 19% and more than 50% after intralesional curettage [8].

The monoclonal antibody denosumab is a recent systemic treatment option for GCTB. It acts by binding to and inhibiting the receptor activator of nuclear factor kappa-Β ligand (RANKL), leading to the loss of osteoclasts from bone surfaces and has been approved by the FDA since 2013 for the treatment of GCTB that are difficult to resect. The first study in 2010 showed promising results with an 86 % (30/35) response rate based on histological and radiological examinations without detectable progression of disease under denosumab treatment [10]. A second larger study demonstrated a stop of disease progression in 69 % of patients with unresectable GCTB after a median of 13 months of denosumab therapy. 74/100 patients in this study with resectable GCTB did not need surgical treatment after neoadjuvant treatment of denosumab, 16/26 were operated less invasively than previously scheduled [11]. In a multicenter study Rutkowski et al. [12] showed that denosumab is extremely efficient in unresectable/metastatic disease as well as in a neoadjuvant setting. The authors conclude that neoadjuvant therapy with denosumab is the option for treatment of initially locally advanced tumors to facilitate complete surgical resection or avoid mutilating surgery.

Nowadays denosumab treatment in case of GCTB is seen more critically. There is a debate if denosumab administered before curettage may increase the likelihood of local recurrence [13]. Sano et al. [14] demonstrated in a study of 87 patients with GCTB that the use of denosumab prior to curettage possibly increase the risk of local recurrence. Tsukamoto et al. [13] conclude in a systematic review that neoadjuvant denosumab application may increase the likelihood of local recurrence after intralesional curettage of GCTB.

This study investigates the risk of local recurrence as well as the recurrence-free survival in patients with GCTB appreciating the use of denosumab and investigates potential risk factors for local recurrence or treatment related complications.

## 2 Materials and methods

The study protocol for this study was approved by the regional ethics committee (reference no.: 2021–804-f-S).

This retrospective study analysed 115 cases of GCTB which were treated at a tertiary academic referral center for orthopedic oncology from 2009 to 2019. Epidemiological data, radiographic and histological examinations, different surgical techniques, complications and local recurrence were reviewed. Imaging studies at time of presentation, including radiographs and MRI-scans were analysed in each case and lesions were classified according to Campanacci [15]. The diagnosis was made by histological and immunohistological examinations by expert pathologists.

**Fig. 2:**
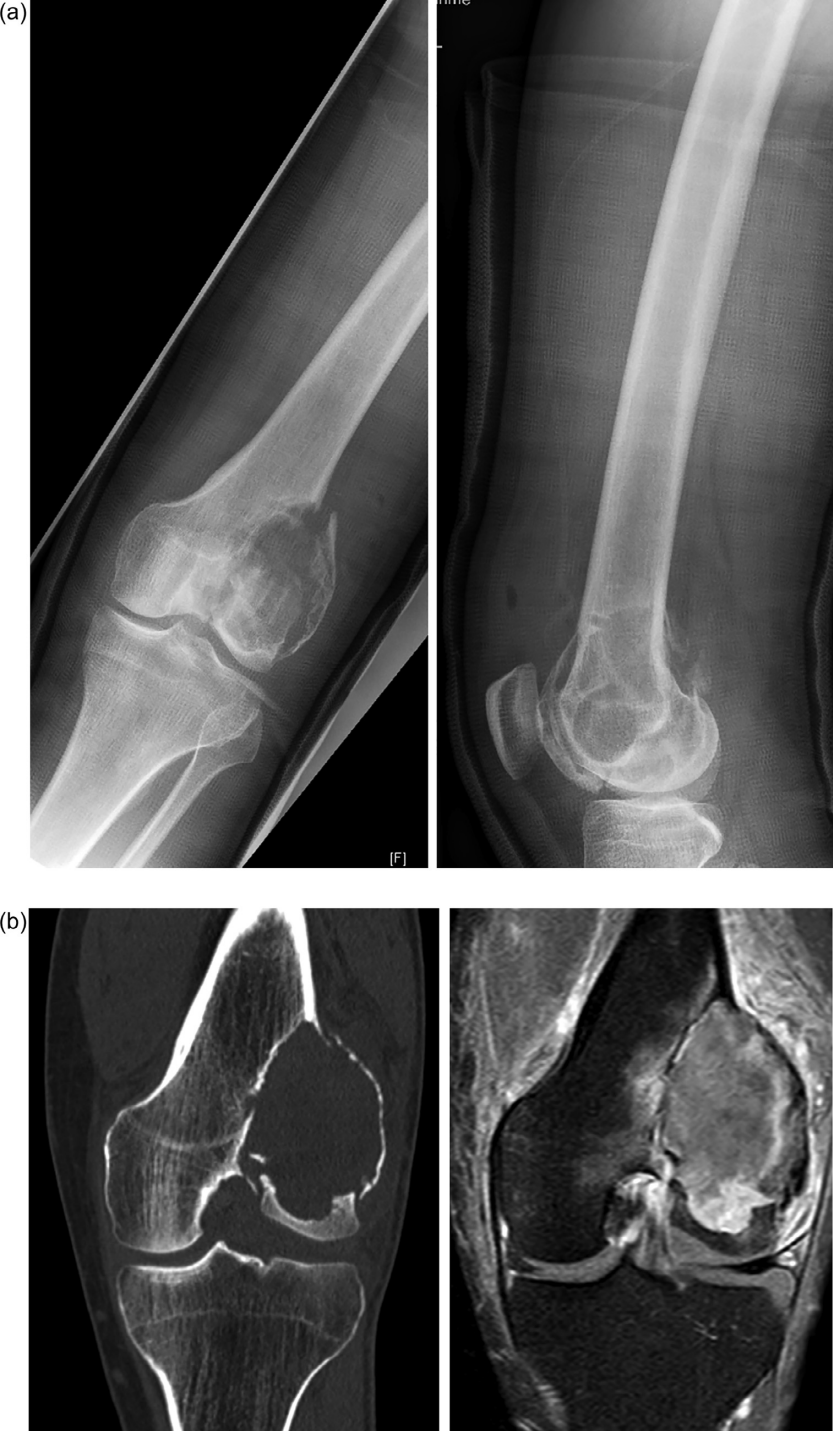

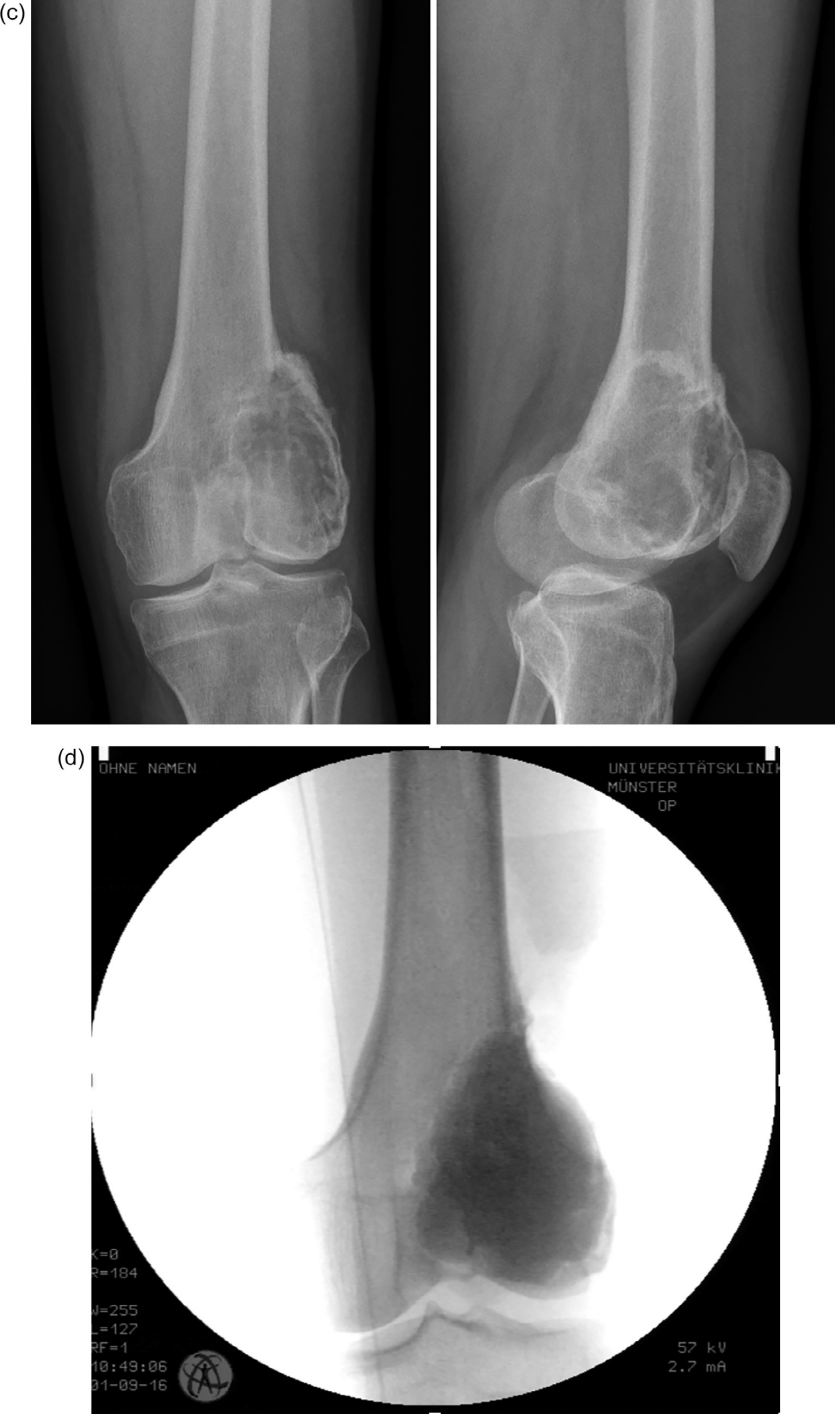

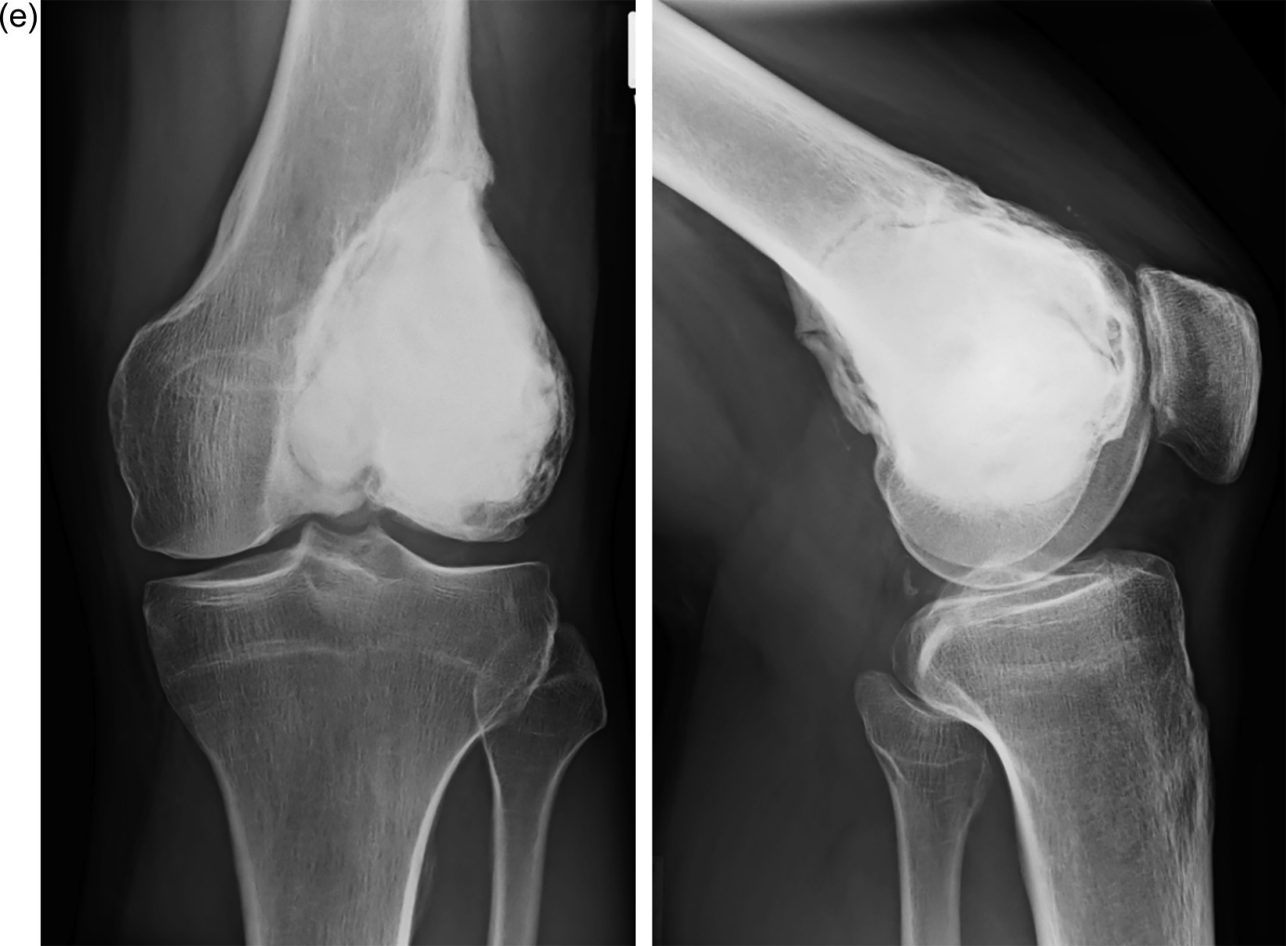
**a-e:** case of a 36-year-old male patient with GCTB of the distal femur with pathological fracture: 2a: radiographs of the distal femur showing an osteolysis with pathological fracture of the lateral condyle; 2b: CT- (left) and MRI-scan (right) of the distal femur with pathological fracture due to a GCTB; 2c: radiographs 3 months after systemic denosumab treatment showing partial sclerosis of the lesion and consolidation of the fracture, 2d: intraoperative radiograph after intralesional curettage and defect reconstruction with bone cement; 2e: radiographs 36 months after surgery without local recurrence.

**Fig. 3:**
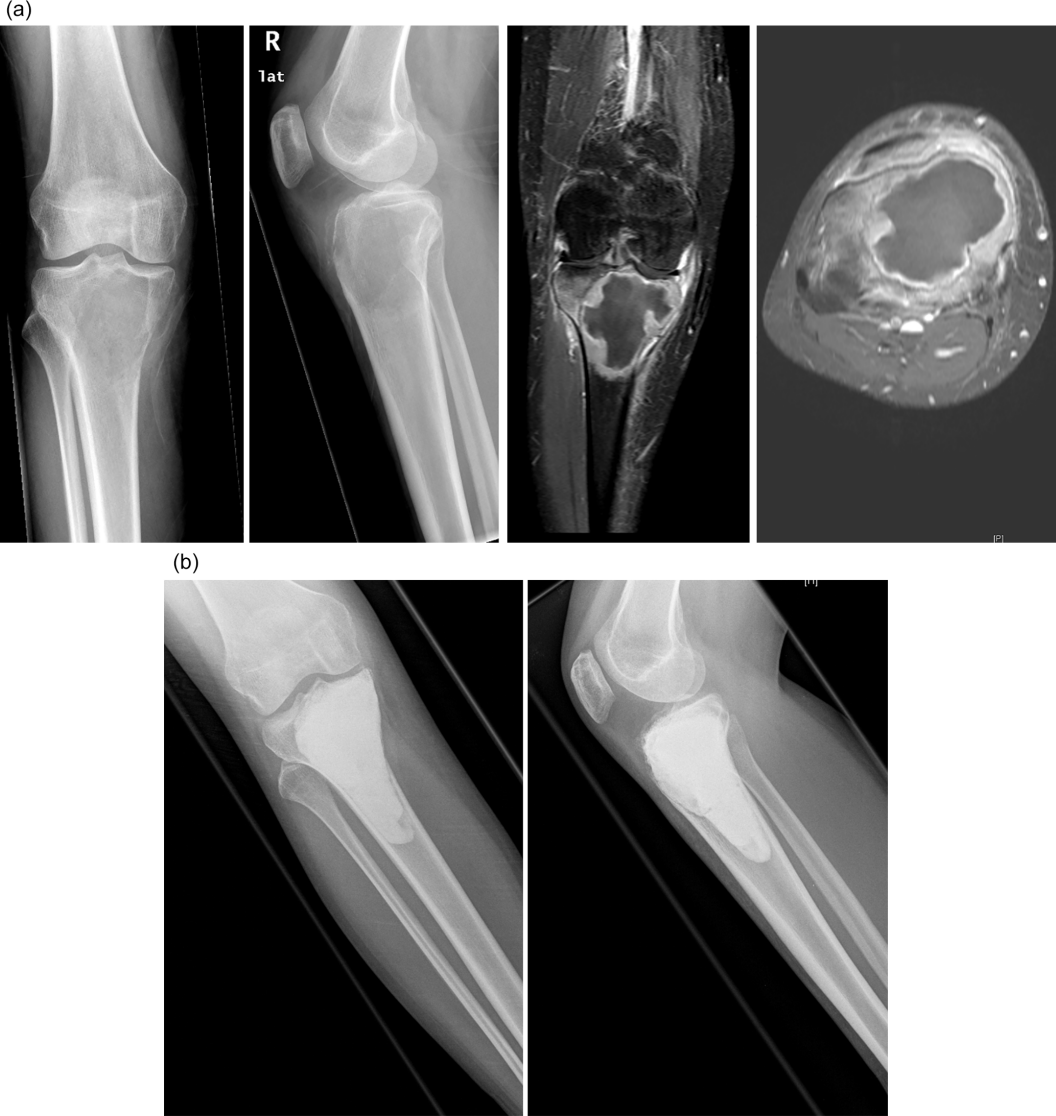

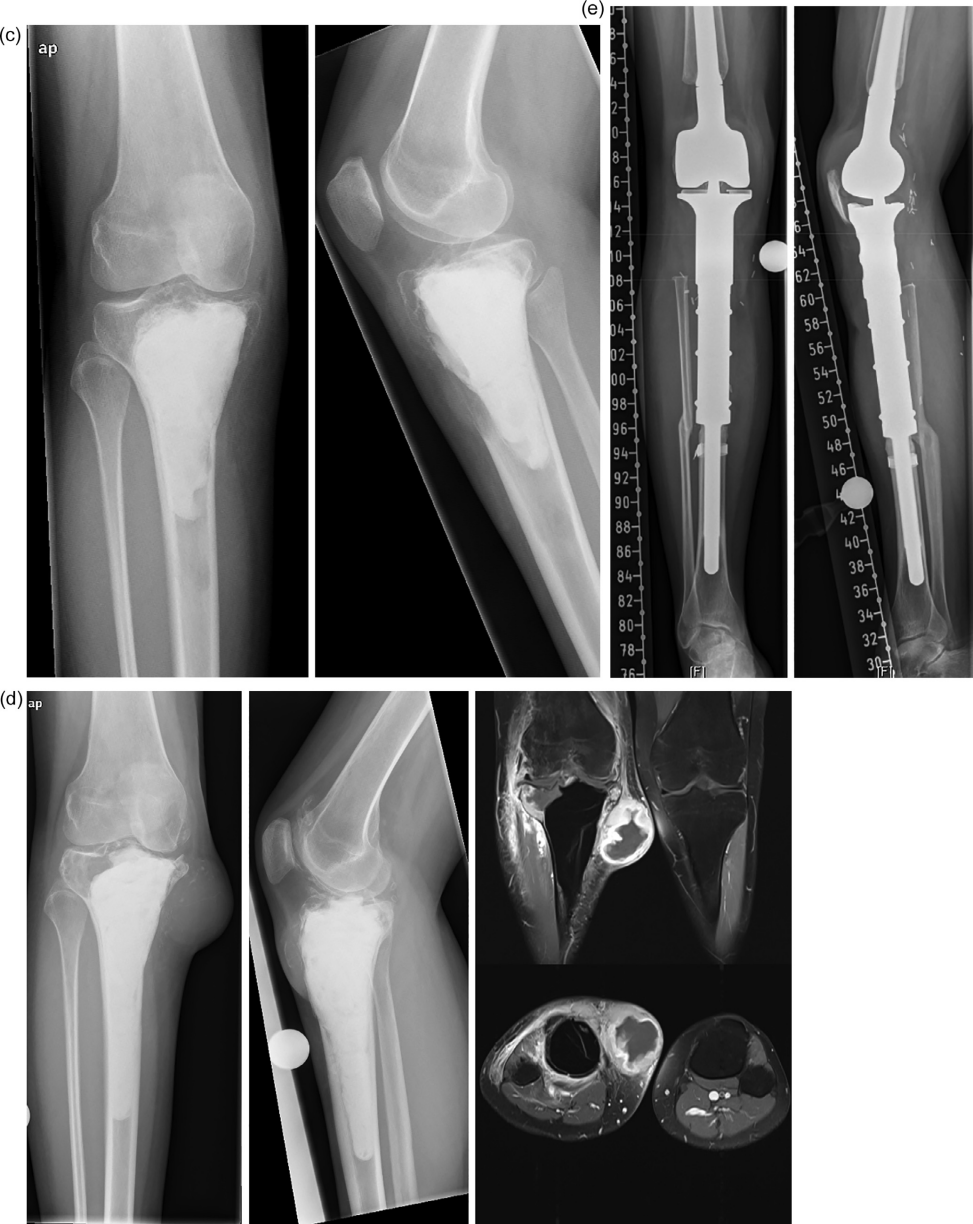
**a-e:** case of a 39-year-old female patient with GCTB of the proximal tibia: 3a: radiographs (left and middle) and MRI-scan (middle and right) of the proximal tibia showing an osteolysis; 3b: postoperative radiograph after intralesional curettage and defect reconstruction with bone cement; 3c radiographs of first local recurrence 18 months after surgery; 3d: radiographs (left and middle) and MRI-scan (middle and right) of second local recurrence 9 months after second curettage; 3e: radiographs of a modular tumor endoprosthesis which had to be implanted due to massive bone defect.

Patients underwent follow-up with clinical and radiographic examinations (radiographs and MRI-scans) at three-month intervals in the first two years and at six-month intervals for the following four years. The therapeutic approach of choice for the majority of all newly diagnosed GCTB was intralesional curettage and defect reconstruction with acrylic bone cement (Palacos®; Heraeus Medical; Wehrheim, Germany). This was sometimes combined with bone substitute (Actifuse®; Baxter Deutschland; Unterschleißheim, Germany) in cases with affection of the subchondral bone juxta articular. In rare cases autologous bone graft or bone substitute were used without additional bone cement for defect reconstruction. All intralesionally resected lesions underwent thorough curettage (Fig. 2a-e) using a high-speed burr as well as careful irrigation with hydrogen peroxide. In cases that would not allow for (repeat) intralesional curettage, infiltration of the joint surface or pathological fracture affecting an adjacent joint, wide resection and modular tumor endoprosthetic replacement (Fig. 3a-e) were performed. Patients with prior interventions elsewhere were excluded from the study. The use of denosumab was reviewed by the local tumor board and was recommended in cases that were not considered resectable, such as Campanacci III-lesions with extensive soft tissue component or intralesional curettage would be facilitated. In these cases, neoadjuvant denosumab was administered for three months preoperatively. Patients received subcutaneous injections of 120 mg denosumab (Xgeva; Amgen, CA, USA) every 4 weeks, with additional doses on days 8 and 15 of the first cycle. All patients received three cycles of treatment with supplements of calcium (500 mg/day) and vitamin D (400 IU/day). A dental examination prior to the first application of denosumab was obligatory. Furthermore, in cases of local recurrence that would not be resectable or result in a mutilating procedure with severe loss of function, denosumab therapy was recommended individually.

Short- or long-term complications, especially of the denosumab therapy were analysed. Digital patient records from the clinical workplace system “ORBIS” (Agfa HealthCare GmbH) as institutional database were reviewed to collect the necessary data.

Statistical analysis was performed with the use of SPSS software, Version 26 (IBM, Armonk, New York, USA). Continuous variables like age and time of follow up were described using the mean and the range. Recurrence rates were analyzed by chi-square test with odds ratios and correlations between relevant risk factors and local recurrence. Cumulative recurrence-free survival was evaluated by Kaplan-Meier analysis with log-rank testing to determine significant differences.

## 3 Results

This study includes 115 patients with the diagnosis of a GCTB. Out of these, 47 patients were male (40.9 %, Table 1) and 68 female (59.1 %, Table 1); the mean age (Table 1) was 33.9 (10–77) years. The mean follow-up time was 65.6 months (24–404). The most common location of manifestation was the distal femur (43; 37.4 %), followed by the proximal tibia (28; 24.3 %) and the distal radius (9; 7.8 %). In six cases the proximal femur was affected (5.2 %), in five cases (4.3 %) the foot and in 3 cases (2.6 %) the pelvic region. According to the Campanacci classification three (2.6 %) lesions were grade 1, 32 (27.8 %) grade 2 and 80 (69.6 %) grade 3.

**Table 1:** The clinicopathological characteristics of the study group and association with local recurrence.

**characteristics**		**odds ratio**	**95% confidence intervall**	**p-value**	**correlation coefficient**	**p-value**
**age**		1.006	0.980–1.033	p = 0.645	ε = 0.043	p = 0.642
	Age less than 18 years	0.480	0.153–1.506	0.209	φ = 0.119	0.202
	Age greater than 18 years	2.082	0.664–6.528	0.209	---	---
**gender**					φ = 0.004	p = 0.966
	female	0.984	0.468–2.070	0.966		
	male	1.016	0.483–2.138	0.966		
**pathological fracture**					φ = 0.139	p = 0.135
	no	2.771	0.696–11.032	0.148		
	yes	0.361	0.091–1,436	0.148		
**location (proximal/distal)**					φ = 0.367	p = 0.367
	distal	0.698	0.320–1.525	0.368		
	proximal	1.432	0.656–3.129	0.368		
**location (upper/ lower extremity)**					φ = 0.035	p = 0.709
	lower	0.835	0.324–2.153	0.709		
	upper	1.198	0.465–3.088	0.709		
**Campanacci classification**					Kramer’s φ = 0.058	p = 0.936
	I	0.500	0.044–5.737	0.578		
	II	1.765	0.145–21.474	0.656		
	III	2.00	0.174–22.949	0.578		
**neoadjuvant use of denosumab**					φ = 0.154	p = 0.101
	no	2.708	0.796–9.211	0.111		
	yes	0.369	0.109–1.256	0.111		
**metastases**					φ = 0.1	p = 0.356
	yes	3,283	0.331–32.537	0.310		
	no	0.305	0.031–3.019	0.310		
**secondary ABC**					φ = 0.035	p = 0.704
	no	0.852	0.374–1943	0.704		
	yes	1.173	0.515–2.676	0.704		
**joint affection**					φ = 0.169	p = 0.069
	no	0.335	0.098–1.138	0.080		
	yes	2.989	0.879–10.164	0.080		
**extraosseous component**					φ = 0.109	p = 0.617
	no	0.713	0.320–1.588	0.408		
	yes	1.402	0.630–3.120	0.408		

In 31 cases (27.0 %) the histologically diagnosed GCTB was associated with a secondary aneurysmal bone cyst. Joint involvement occurred in 14 patients (12.2 %). In all cases plain radiographs or CT scans of the lung were performed which revealed pulmonary metastases in four cases (3.5 %).

In 14 cases (12.2 %) a preoperative denosumab therapy was initiated. The length of the preoperative therapy was 12 weeks. In 14.8 % (17 cases) of the patient group denosumab was applied in case of local recurrence preoperatively. Thus, Campanacci grade III-lesions with extensive soft tissue became operable. In two cases (1.7 %) patients were treated with denosumab without following surgery. Complications of the denosumab therapy were jaw necrosis (3/33; 9.1 %) and polyarthralgia with myalgia in three cases (3/33; 9.1 %), respectively.

As most common treatment intralesional curettage (Fig. 2a-e) was performed in 105 cases (91.3%). In every case hydrogen peroxide was used as adjuvant (100%), in 88 cases (76.5%) the resulting cavity was filled with bone cement, in 7 cases (6.1 %) with bone substitute and in 10 cases (8.7%) with autogenous bone graft. Four patients (3.5%) were treated with a wide resection and implantation of a modular tumor endoprosthesis (Fig. 3a-e), in one case (0.9%) a hemipelvectomy was necessary for tumor resection. Three patients were treated by simple observation due to the lack of symptoms without progression of the lesion.

Intraoperative complications occurred in two cases in form of a remaining fragment of bone cement in the affected joint resulting in an arthroscopic removal. In four cases (3.5%) lung metastasis were found.

Analysing the results of local recurrence, the following differences were found: the overall recurrence rate was 47.8 % (55/115 cases), in the subgroup with intralesional curettage, use of hydrogen peroxide as adjuvant and defect reconstruction with bone cement without neoadjuvant denosumab application 42.2 % (38/90 cases) and in the subgroup with neoadjuvant denosumab application and intralesional curettage 28.6 % (4/14 cases). After wide resection and endoprosthetic replacement one case (20%) of local recurrence was detectable (1/5 cases). In case of the first local recurrence a re-curettage with additional preoperative denosumab therapy of 12 weeks resulted in a high recurrence rate of 50 % (8/16 cases). In the subgroup without denosumab treatment the rate of second local recurrence after initial curettage was 33.3% (11/33 cases). However, the log-rank test did not show any statistically significant difference (p = 0.214) for the two subgroups. The mean follow-up time until local recurrence arose was 20.1 (2–117) months in the curettage group without denosumab and 26 (3–86) months in the curettage group with denosumab. Locations with the highest local recurrence rate after initial curettage were the distal femur 30.9 % (17 patients) and the proximal tibia 29.1 % (16). There was no correlation between age (p = 0.642), gender (p = 0.966), localization (p = 0.762), pathological fracture (p = 0.148), Campanacci grade (p = 0.578–0.656), metastases (p = 0.310), secondary aneurysmal bone cyst (p = 0.704), joint affection (p = 0.08), and local recurrence of GCTB, respectively. Analysing the recurrence-free survival time between the curettage group with and without neoadjuvant denosumab treatment, no significant difference was found (p = 0.714). The standard surgical treatment after initial curettage in case of local recurrence was a re-curettage with partial or complete resection of the cement filling in 58.2 %. In four cases an endoprosthesis hat to be implanted (Fig. 3a-e).

## 4 Discussion

This study aimed to compare the local recurrence of patients with GCBT after different surgical treatments. Additionally, it comments on the question if denosumab influences local recurrence. The number of cases treated by intralesional curettage without neoadjuvant denosumab (n = 82) treatment was significantly higher than the number of patients with wide resection (n = 4) or preoperative denosumab treatment (n = 31).

Regarding the epidemiology, GCTB occurs more often in female than in male patients [2]. In our study male patients represent 40.9 %, females 59.1 % of the collective; the mean age is 33.9 (10–77) years. This is according to other studies where a peak age of 20 – 40 years is reported [5], [16]. In literature most common locations for GCTB manifestation are the distal femur, the proximal tibia and the distal radius [2], [5]. In analogy our results indicate the distal femur (43; 37.4 %), followed by the proximal tibia (28; 24.3 %) and the distal radius (9; 7.8 %) as most common locations. Localization is often described in metaepiphyses [17]. In our study the majority of cases were localized in the epiphyses with joint affection in 14 patients (12.2 %).

Surgical treatment in terms of an intralesional curettage with or without the use of adjuvants is the most common treatment for GCTB [2], [7], [8]. In this study an intralesional curettage was performed in 90 cases, combined with the use of a high-speed burr, hydrogen peroxide and bone cement for defect reconstruction. Gao et al. [18] reported about a local recurrence rate of 13% after intralesional curettage with bone cement as adjuvant in case of GCTB. In a second subgroup of this study without cement augmentation the recurrence rate was significantly higher (35%). Balke et al. [2] demonstrated that the combination of different adjuvants (use of a high speed burr, hydrogen peroxide and bone cement for defect reconstruction) reduced the likelihood of local tumor recurrence by the factor 28.2 compared to intralesional curettage only. Nevertheless, the benefit from hydrogen peroxide did not reach statistical significance. Subsequently, Omlor et al. [7] proved for the first time that the additional use of hydrogen peroxide as adjuvant in case of intralesional curettage of GCTB significantly increased recurrence-free survival in a study of 51 patients. The authors reported a recurrence rate of 41% after intralesional curettage with the use of hydrogen peroxide. In other studies, the local recurrence rate of GCTB after intralesional curettage ranged from 13% − 50% [8], [18], [19], [20]. In the present study the local recurrence rate after the standard treatment of intralesional curettage with the use of a high-speed burr, hydrogen peroxide as adjuvant and defect filling with bone cement was 42.2% (38/90 cases) and thus comparably high. In 10 cases bone substitute was combined with bone cement for defect reconstruction if the tumor reached close to the articulating surface. Balke et al. [2] showed that a subchondral bone graft did not provoke a higher local recurrence in a study of 214 patients with GCTB treated mainly by intralesional curettage and cement augmentation.

For various clinicopathological factors like age, gender or localization of GCTB but even for joint affection or the presence of metastases no significant association with local recurrence was found in the recent study. Sano et al. [14] did not find any association between various clinicopathological factors such as age, gender, surgical site, Campanacci grade or pathological fracture and local recurrence either in a study of 87 patients with GCTB.

After wide resection a significantly lower recurrence rate of 20% (1/5 cases; p = 0.01) compared to intralesional curettage could be detected. Due to the low number of patients in this subgroup the results must be interpreted cautiously. Liu et al. [21] reported about a low local recurrence rate of 6% after wide resection of GCTB in a small comparative study of 27 patients. Tuntarattanapong [22] reached a local recurrence rate of 2.4% in a study with 41 patients and wide resections of GCTB.

After systemic application of the monoclonal antibody denosumab as neoadjuvant treatment option and intralesional curettage the recurrence rate after was 28.6 % (4/14 cases) in the present study. However, the group size of this subgroup is limited, and the statistical analysis showed a non-significant difference (p = 0.714) compared to the subgroup of patients treated by intralesional curettage without denosumab application. Nevertheless, previously borderline resectable lesions became operable after three months of neoadjuvant treatment with denosumab (Fig. 2a-e). Traub et al. [23] reported a local recurrence rate of 17% with a median follow-up of 30 months in a study of 20 patients who received denosumab for 6–11 months in a neoadjuvant setting before intralesional curettage. Thus, the local recurrence rate was comparable to other studies without preoperative denosumab treatment [2]. Errani et al. [24] demonstrated a high local recurrence rate of 60% (15/25) in a cohort of patients treated with denosumab and subsequent intralesional curettage. The control group of 222 patients had a recurrence rate of 16% (36/222). Other studies confirmed this impression. Agarwal et al. reported a recurrence rate of 44% (11/25) after intralesional curettage with preoperative denosumab treatment compared to 21% (7/34) in the control group without denosumab application, although it was not statistically significant. In a recent review including seven studies with 619 patients Tsukamoto et al. [13] found a proportion of patients with local a recurrence rate ranging from 20% to 100% in the curettage group with preoperative denosumab treatment and a local recurrence ranging from 0% to 50% in the curettage-alone group. In order to explain the results, the authors put forward the hypothesis that the newly formed bone on the periphery of the lesion may harbour neoplastic cells that may reactivate in case of recurrence when the shell is not completely excised during curettage [23]. Additionally, in our experience the newly formed bone makes curettage more effortful and thus prolongates operating times. However, in the present study denosumab did not increase the risk of local recurrence after preoperative treatment and first intralesional curettage, although the lesions of this subgroup were primarily classified as borderline resectable lesions without neoadjuvant treatment. In contrast, the recurrence rate after preoperative denosumab treatment and intralesional curettage was lower compared to the subgroup without denosumab application (26.6 vs 42.2%; p = 0.714).

On the other hand, in the subgroup with local recurrence after (first) intralesional curettage an additional positive effect of denosumab before re-curettage was not detectable. In 50 % (8/16 cases) patients had a second recurrence after the first recurrence treated by preoperative denosumab therapy and intralesional re-curettage. The log-rank test did not show a statistically significant difference (p = 0.214) for the second local recurrence between the subgroups with (50%) and without (33.3%) preoperative denosumab treatment before re-curettage. In a comparable study of 87 patients with GCTB Sano et al. report about a recurrence rate of 80% after re-curretage and preoperative denosumab application in case of local recurrence.

The main complications of denosumab treatment in the present study were jaw necrosis and reduction of general conditions in three cases (3/33; 9.1 %), respectively. Due to these complications the treatment was stopped in all six cases. Palmerini [25] evaluated the toxicity of denosumab in a retrospective series of 97 patients who were treated for a median of 12 months. Overall, six patients (11%) developed an osteonecrosis of the jaw and patients on prolonged treatment a mild peripheral neuropathy (6/54; 11%), skin rash (5/54, 9%), hypophosphatemia (2/54; 4%) and atypical femoral fracture (2/54; 4%). In the largest phase II trial published by Chawla et al. [11] patients were treated up to 13 months with acceptable toxicity and sustained response to denosumab. In rare cases, transformation of benign GCTB to a malignant bone tumor are described in literature [26]. This may happen due to dedifferentiation, prior radiation therapy, misdiagnosis or malignant transformation [8]. Some case reports were published reporting a malignant transformation under denosumab therapy [27], [28], [29]. However, the above-mentioned phase II trial of Chawla et al. [30] revealed only one case of malignant transformation in a cohort of 282 patients treated by denosumab for up to 13 months. No sign of malignant transformation was found in GCTB of patients of the present study.

This study has several limitations; it is a retrospective, not randomized study with a limited number of patients. The number of cases treated by intralesional curettage without neoadjuvant denosumab treatment was significantly higher than the number of patients with wide resection or preoperative denosumab treatment. In addition, only patients with borderline resectable lesions received a neoadjuvant denosumab treatment (selection bias). Thus, the comparative statistical analysis has to be interpreted cautiously. We agree to Gaston et al. [31] that larger randomized studies are necessary to confirm the above motioned results and to answer the question if local recurrence rates will be decreased with the adjuvant use of denosumab along with surgery.

## 5 Conclusion

Therapeutically, an aggressive intralesional curettage with hydrogen peroxide as adjuvant and filling up the defect with bone cement leads to considerable recurrence rates but is nevertheless a justifiable treatment. Preoperative denosumab therapy in patients with GCTB does not increase significantly local recurrence in this study. Nevertheless, the use of denosumab in GCTB should be limited to cases with high surgical morbidity and for restricted time.

## 6 Ethical review committee statement

The study protocol was approved by the regional ethics committee (reference no.: 2021-804-f-S).

## 7 Type of study

Level IV study, retrospective case series.

## Funding

The APC was funded by the Open Access Publishing Funds of the Westphalian Wilhelms University of Muenster.

## Declaration of Competing Interest

The authors declare that they have no known competing financial interests or personal relationships that could have appeared to influence the work reported in this paper.
